# Alkaline Soil pH and Phosphorus Depletion Are Associated with Microbial Community Changes Under Long-Term Continuous Cropping of *Paeonia ostii*

**DOI:** 10.3390/microorganisms14102248

**Published:** 2026-10-03

**Authors:** Xin Tang, Ruiming Bi, Yingying Li, Bowen Wu, Lingfu Kong, Menglu Guo, Xiuyan Yang, Li Zhang

**Affiliations:** 1 College of Agricultural and Biological Engineering, Heze University, Heze 274015, China; tangxin@hezeu.edu.cn (X.T.); biruiming@hezeu.edu.cn (R.B.); lyying80@163.com (Y.L.); konglingfuu@163.com (L.K.); guomenglu@hezeu.edu.cn (M.G.); 2 Heze Academy of Forestry, Heze 274099, China; wu_bowen0813@163.com; 3 Institute of Ecological Conservation and Restoration, Chinese Academy of Forestry, Beijing 100091, China; 4 Tianjin Institute of Forestry Science, Chinese Academy of Forestry, Tianjin 300457, China; 5 Comprehensive Experimental Center of Chinese Academy of Forestry in Yellow River Delta, Dongying 257500, China

**Keywords:** soil bacterial diversity, soil fungal diversity, continuous cropping, rhizosphere soil, *Paeonia ostii ‘Feng Dan*’

## Abstract

Long-term continuous cropping often degrades soil and reduces productivity, yet how soil properties and microbial communities respond in continuous tree peony monoculture remains poorly understood. Herein, soil physicochemical properties were investigated across a monoculture chronosequence of 1, 3, 5, 8, and 10 years of continuous tree peony cropping, together with rhizobacterial and fungal community diversity, composition, and predicted functions. The contents of soil organic matter, total nitrogen and available nitrogen increased along the chronosequence, whereas the contents of available phosphorus declined sharply after 3 years and remained low thereafter. Bacterial richness declined monotonically, whereas fungal richness reached its lowest level at 8 years. Beta-diversity revealed a clear community succession, with *Acidobacteriota* suppressed, and fungal communities shifted directionally from *Fusarium* to *Phoma* and ultimately to *Monographella* in the 10-year fields. Predicted functional profiling suggested a potential metabolic shift from energy-intensive pathways to stress-adaptive processes, including enhanced membrane transport. Redundancy analysis and structural equation modelling further indicated that pH and available phosphorus were strongly associated with microbial assemblages, with monoculture duration indirectly linked to communities through alterations in these soil properties. Overall, phosphorus availability, rather than bulk organic matter accumulation, emerged as the primary factor shaping microbial community dynamics and pathogen succession in this alkaline agroecosystem. These findings point to stage-specific pathogen control combined with phosphorus optimization as a potential strategy to mitigate replant disease in tree peony monoculture systems.

## 1. Introduction

*Paeonia ostii* ‘Feng Dan’ is a tree peony cultivar valued for its high seed productivity and edible oil production [1,2]. With expanding cultivation and prolonged monoculture, tree peony plants exhibit progressive growth decline, reduced flowering and seed set, heightened susceptibility to pests and diseases, and even widespread mortality. This phenomenon has emerged as a critical constraint to the sustainable development of the tree peony oil industry.

Continuous cropping obstacles (replant disease) are closely linked to soil microenvironment alterations, including microbial community imbalances, root exudate accumulation, and enzyme activity changes [3,4,5,6,7,8]. Microbial imbalances typically involve a decline in beneficial taxa and an enrichment in potential pathogens, which can disrupt key ecosystem functions such as nutrient cycling and disease suppression [4,5,6,7]. For instance, In *Paeonia ostii*, five phenolic acids—ferulic acid, cinnamic acid, vanillin, coumarin, and paeonol—have been detected in the rhizosphere soil and shown to inhibit seedling growth and root development [3]. The accumulation of such autotoxic compounds can alter root exudate profiles and create a selective pressure that favors pathogenic taxa while suppressing beneficial microbes [4], leading to a dysbiotic rhizosphere microbiome [5,6,7], as root exudates can act as selective carbon sources and signaling molecules that shape microbial community composition [4]. In parallel, continuous cropping has been shown to affect soil enzyme activities, with reduced urease and sucrase activities and increased cellulase activity reported in *Astragalus membranaceus*, reflecting impaired nutrient cycling and soil functional degradation [8]. These combined effects—shifts in microbial community structure, accumulation of autotoxic compounds, and altered enzyme activities—contribute to nutrient depletion, disruption of microbial ecological balance [9,10], and ultimately, soil degradation and replant problems [8,11]. However, findings from previous studies on microbial responses to continuous cropping remain inconsistent. For example, long-term monoculture of *Salvia miltiorrhiza* [12] and potato [13] altered rhizosphere fungal and actinomycete communities, while continuous cropping of sweet potato was reported to reduce bacterial diversity [7,14] but increase fungal diversity [6]. In *Panax notoginseng*, both bacterial and fungal communities differed significantly between healthy and root-rot plants [5,15]. For tree peony, Pan et al. [16] examined soil properties and bacterial communities across 1-, 4-, and 10-year chronosequences, but their three time points and the absence of fungal data leave the co-occurring dynamics of both bacterial and fungal assemblages largely unexplored. Consequently, the successional trajectories of rhizosphere microbial communities under continuous tree peony cropping remain poorly understood. To address these gaps, this study combined high-throughput sequencing with soil physicochemical analyses to characterize bacterial and fungal community dynamics across a chronosequence of 1, 3, 5, 8, and 10 years of *Paeonia ostii ‘Feng Dan’* monoculture. Specifically, the objectives of this study were as follows: (i) We aimed to investigate variations in soil physicochemical properties along the chronosequence. (ii) We also aimed to characterize the successional patterns of rhizosphere bacterial and fungal community diversity, structure, and composition using 16S rRNA and ITS amplicon sequencing. (iii) We sought to explore potential shifts in microbial metabolic pathways and functions and to identify the soil factors most strongly associated with these microbial changes.

## 2. Materials and Methods

### 2.1. Study Area and Soil Sampling

The experimental soil was collected from the Caozhou Peony Garden (35°14′ N, 115°26′ E) in Heze, Shandong Province, China, a major cultivation area for tree peonies. The site has a contiguous planting area of approximately 106 ha and is characterized by a warm-temperate monsoon climate, with a mean annual temperature of 11–14 °C and annual precipitation of 550–950 mm.

Soils of oil peony (*Paeonia ostii* ‘Feng Dan’) were collected from fields with different planting durations: established in 2022 (1 year, Y1), 2020 (3 years, Y3), 2018 (5 years, Y5), 2015 (8 years, Y8), and 2013 (10 years, Y10). Sampling was conducted during the seed harvest period in August 2023. All plots were located within the same plantation, with uniform soil parent material (classified as moderately alkaline sandy soil) and consistent water–fertilizer management practices. The fertilization regime for *Paeonia ostii* ‘Feng Dan’ included an annual application of organic fertilizer derived from fermented peony seed cake, seed hulls, and fruit shells, applied at a rate of approximately 100 kg ha^−1^.

For each planting duration, three independent composite samples were collected from the field. Each composite sample consisted of five soil cores collected from the 0–20 cm soil layer adjacent to the roots of *Paeonia ostii* ‘Feng Dan’ within the plot and pooled together. Immediately after collection, large rocks and roots were removed by passing the soil through a 2 mm sieve in the field. In total, fifteen composite samples were analyzed in this study (five cropping durations × three independent composite samples) [7]. The samples were promptly transported to the laboratory, where they were sieved again and divided into two portions. One portion was stored at −80 °C for subsequent DNA extraction, while the other was stored at 4 °C for analyses of soil properties and enzyme activities.

### 2.2. Chemical Analysis

Soil pH was measured in a suspension of soil and water (1:2.5 *w*/*v*) using a glass combination electrode [17]. Total nitrogen (TN) was tested via Kjeldahl digestion [18] followed by quantification using a scalar continuous flow analyzer. The molybdate-blue method was used for colorimetric measurement of total phosphorus (TP) at 440 nm [19]. Flame photometry was used to assess the accessible potassium (AK) and total potassium (TK) contents in soil [17]. Available nitrogen (AN) was measured by a micro-diffusion technique after alkaline hydrolysis [20,21]. Available phosphorus (AP) content was determined using the Olsen-P method following the procedures specified for forest soils (LY/T 1232-2015) [22,23]. The total soil organic matter (SOM) was determined by dichromate oxidation [24]. All chemical analyses were performed in triplicate for each composite sample by a certified commercial laboratory following standard protocols. Quality control parameters (recovery rates, accuracy, LOD, and LOQ) were all within acceptable ranges (Table A1). All soil chemical analyses were performed using standard laboratory equipment (detailed instrument information, including model numbers, manufacturers, and countries of origin, is provided in Table A2).

### 2.3. DNA Extraction and Amplification

Total soil microbial DNA was extracted using the CTAB method [25]. DNA integrity and concentration were assessed by agarose gel electrophoresis and Qubit 2.0 Fluorometer (Thermo Fisher Scientific, Waltham, MA, USA); soil microbial DNA was stored at −20 °C. PCR amplification of bacterial 16S V4 and fungal ITS1 regions was performed using primer sets 314F (5′-CCTACGGGNGGCWGCAG-3′) and 806R (5′-GGACTACHVGGGTATCTAAT-3′) and ITS1F (5′-CTTGGTCATTTAGAGGAAGTAA-3′) and ITS2R (5′-GCTGCGTTCTTCATCGATGC-3′) [26,27,28]. Each 25 µL reaction contained 12.5 µL of Phusion^®^ Master Mix (New England Biolabs, lpswich, MA, USA), 0.2 µM of each primer, and 10 ng of template DNA. Thermal cycling was 98 °C for 30 s; 30 cycles of 98 °C for 10 s, 55 °C for 30 s, and 72 °C for 30 s; and 72 °C for 5 min. PCR products were confirmed by agarose gel electrophoresis, purified using magnetic beads, and pooled in equimolar concentrations. Libraries were constructed using the TruSeq^®^ DNA PCR-Free Kit (Illumina, San Diego, CA, USA) and sequenced on an Illumina NovaSeq 6000 platform (Illumina, San Diego, CA, USA) following the manufacturer’s protocols.

### 2.4. Bioinformatics Analysis

Raw reads were filtered and trimmed using QIIME v.1.9.1 [29], and sequences were retained within the 220–500 bp range. High-quality sequences were processed using Mothur v.1.34.4 [30], and chimeras were removed using USEARCH [31]. OTUs were clustered at 97% similarity. Alpha diversity indices were calculated using QIIME v.1.9.1. Taxonomic assignment was performed using SILVA v.138 SSU rRNA database [32] and UNITE databases v.8.2 [33]. All statistical analyses were performed in R v.4.3.1 (R Core Team, 2024) [34]. Prior to analysis of variance (ANOVA), centered log-ratio-transformed (CLR-transformed) data [35] were tested for normality (Shapiro–Wilk test) and homogeneity of variances (Levene’s test) [36,37]. One-way ANOVA with post hoc comparisons using Tukey’s HSD test (for equal variances) or Games–Howell test (for unequal variances) was applied to CLR-transformed relative abundance data [38,39]. PERMANOVA (999 permutations) was performed based on Bray-Curtis dissimilarity matrices calculated from Hellinger-transformed species abundance data to test for differences in community composition among cropping durations [40,41]. Prior to PERMANOVA, homogeneity of multivariate dispersion was tested using PERMDISP (betadisper function in the vegan package) with 999 permutations to confirm that significant PERMANOVA results were not driven by differences in group dispersion [42]. Pearson correlation coefficients were calculated to examine relationships between soil properties and microbial diversity indices. Redundancy analysis (RDA) and distance-based redundancy analysis (db-RDA) were performed to examine the relationships between soil properties and microbial (bacterial and fungal) community composition and functional structure, using the vegan R package (v.2.7.1) [43]. Prior to analysis, species abundance data were Hellinger-transformed, and environmental variables were standardized (centered and scaled). Variance inflation factors (VIFs) were calculated for all soil variables to avoid overparameterization, and variables with VIF > 10 were removed [44]. The significance of the overall models and individual soil variables was assessed using Monte Carlo permutation tests (999 permutations), with individual variable significance evaluated via the envfit function in the vegan package [43]. Functional profiles predicted by PICRUSt2 were used directly in RDA without additional transformation. Venn diagrams were generated using the ggVennDiagram R package [45] to visualize shared and unique taxa among different cropping years. LEfSe (linear discriminant analysis effect size) analysis was performed to identify indicator taxa among cropping durations, and taxa with LDA (linear discriminant analysis) scores > 2 and *p* < 0.05 were considered significantly enriched [46]. Functional predictions were performed using FUNGuild and PICRUSt2 (KEGG) with default parameters following the recommended pipelines [47,48,49]. The confidence of FUNGuild assignments was recorded for each taxonomic assignment, and the distribution of confidence categories (highly probable, probable, possible, and unassigned) was summarized. SEM was performed using IBM SPSS AMOS (v.21.0), with soil variables selected through an iterative process guided by modification indices; model fit was assessed using CFI > 0.95, RMSEA < 0.05, and GFI > 0.90 [50,51].

## 3. Results

### 3.1. Changes in Soil Chemical Properties of Paeonia ostii ‘Feng Dan’ During Different Continuous Cropping Years

ANOVA (Table 1) revealed that cropping duration significantly affected most measured soil parameters (*p* < 0.01). Soil pH peaked at Y3, which was statistically higher than that in Y5, Y8, and Y10 but remained comparable to that in Y1 (*p* > 0.05). TN concentrations remained consistently low during the initial three years (0.73–0.75 g kg^−1^), increased significantly by Y5, and subsequently stabilized at a higher plateau (0.95–1.03 g kg^−1^) from Y5 to Y10. TP varied significantly with planting duration (*F* = 9.03, *p* = 0.002). Post hoc comparisons showed that TP was highest in Y1 and Y8 and significantly lower in Y5 and Y10, with Y3 showing intermediate values that did not differ from either group. TK content showed a modest but significant response to planting duration (*F* = 8.95, *p* = 0.002). AN concentration responded significantly to the planting chronosequence (*F* = 48.05, *p* < 0.002). The AN decreased significantly from Y1 to Y3 and then increased steadily thereafter, with Y8 and Y10 showing the highest values. In contrast, AP declined by approximately 50% between Y3 and Y5 (Table 1). AK also responded significantly to planting duration (*F* = 26.48, *p* < 0.001): AK increased from Y1 to Y5 and Y8 and then stabilized at an intermediate range by Y10. SOM content was significantly influenced by the planting duration (*F* = 111.18, *p* < 0.001), remaining low in the first two years, increasing markedly from Y5, and peaking at Y10. Overall, prolonged monoculture was associated with progressive accumulation of SOM, TN, and AN, alongside a marked decline in AP and a transient peak in pH at Y3.

### 3.2. Sequencing Results and OTUs in the Rhizosphere Soil of Paeonia ostii ‘Feng Dan’ with Different Cropping Durations

High-throughput sequencing of the bacterial 16S rDNA V3-V4 region and fungal ITS region generated 855,133 and 1,203,795 quality-filtered reads, respectively (Table A3). These reads were clustered into 3539 bacterial and 1584 fungal OTUs at a 97% similarity level. Bacterial OTU richness peaked at Y3 (2043 OTUs) and gradually declined thereafter, reaching a minimum at Y10 (1803 OTUs). Fungal richness showed greater variability, with a pronounced decline at Y8 (396 OTUs) followed by recovery at Y10 (546 OTUs).

**Figure 1:**
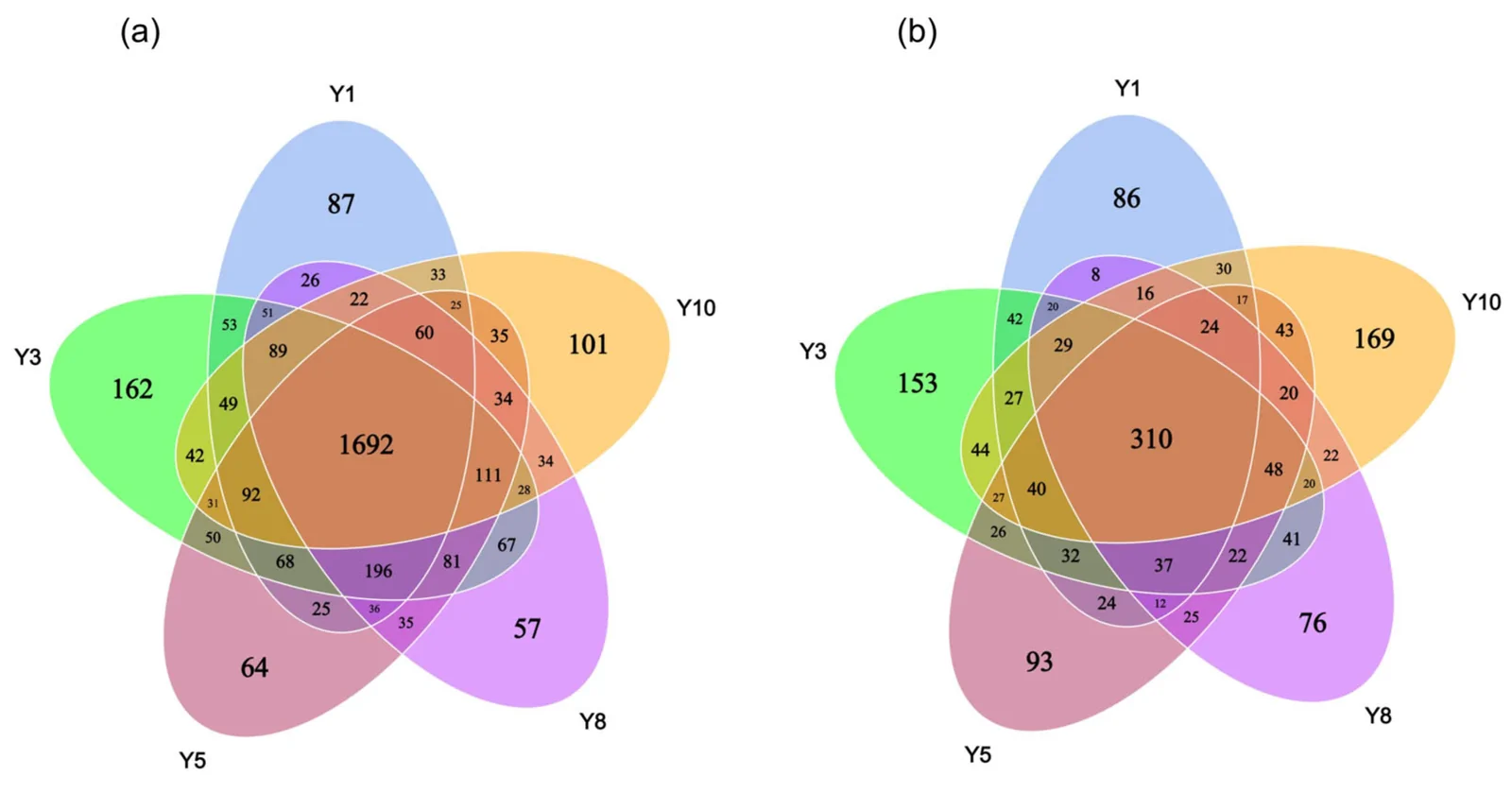
Venn diagrams showing shared and unique OTUs of soil bacterial (**a**) and fungal (**b**) communities across the continuous cropping chronosequence of *Paeonia ostii* ‘Feng Dan’. Explanations: Same as for Table 1.

Venn diagrams were used to visualize the distribution of unique and shared OTUs across groups (Figure 1). For bacteria, 1692 OTUs were shared across all five groups (Figure 1a). The number of unique bacterial OTUs for Y1, Y3, Y5, Y8, and Y10 were 87 (4.59%), 162 (7.92%), 64 (3.32%), 57 (3.01%), and 101 (5.60%), respectively. For fungi, 310 OTUs were common to all groups (Figure 1b). The proportions of shared fungal OTUs relative to total OTUs in Y1, Y3, Y5, Y8, and Y10 were 61.75%, 54.86%, 60.42%, 78.28%, and 56.77%, respectively. The numbers of unique fungal OTUs for Y1, Y3, Y5, Y8, and Y10 were 86 (17.13%), 153 (27.07%), 93 (18.12%), 76 (19.19%), and 169 (30.95%), respectively.

### 3.3. Microbial Community Diversity in the Rhizosphere Soil of Paeonia ostii ‘Feng Dan’ During Different Cropping Durations

**Figure 2:**
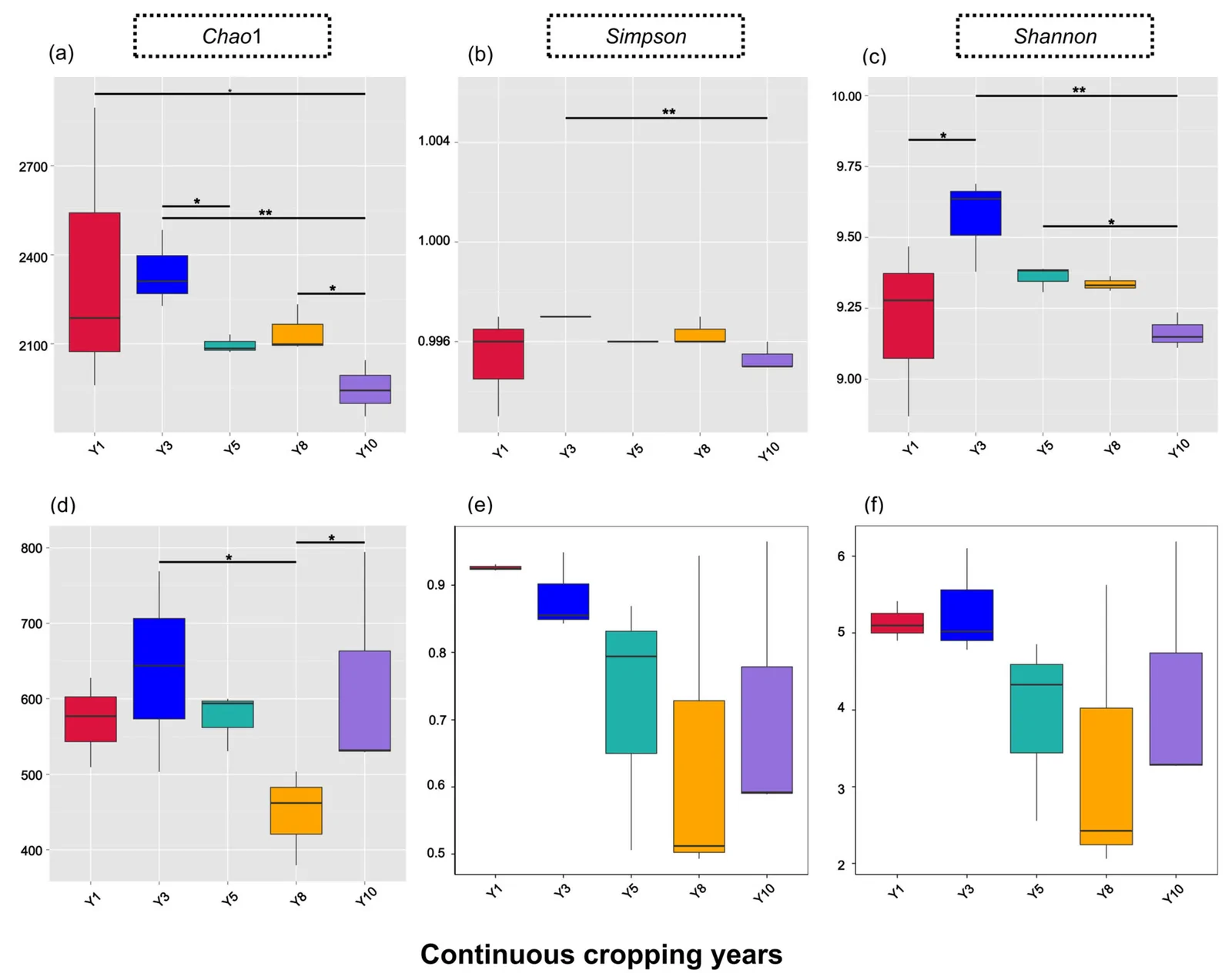
Bacterial diversity (**a**–**c**) and fungal diversity (**d**–**f**) in the rhizosphere soil of *Paeonia ostii* ‘Feng Dan’ across the continuous cropping chronosequence. Statistical significance was determined using the Wilcoxon rank-sum test. * *p* < 0.05; ** *p* < 0.01. Explanations: Same as for Table 1.

**Figure A1:**
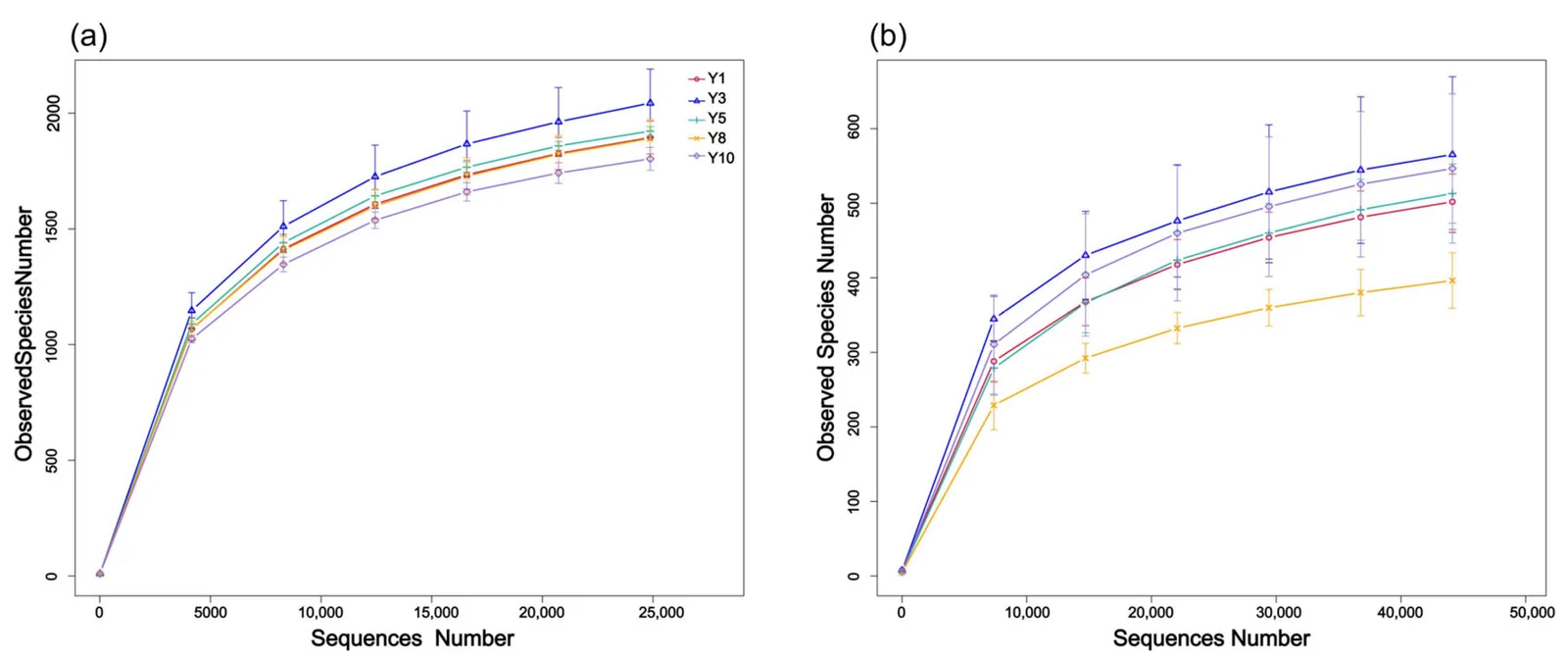
Rarefaction curve of bacteria (**a**) and fungi (**b**) OTUs abundance.

Rarefaction curves confirmed adequate sequencing depth for all samples (Figure A1). For bacterial communities, the Chao1 index decreased after long-term cropping with Y10, showing a 17.04% reduction relative to Y1. The overall trend showed a decline from Y1 to Y5, a slight recovery at Y8, and a further drop at Y10. Significant pairwise differences in Chao1 were detected between Y1 and Y10 (*p* < 0.05), Y3 and Y10 (*p* < 0.05), Y3 and Y5 (*p* < 0.05), and Y8 and Y10 (*p* < 0.01) (Figure 2a; Table A4). Bacterial Shannon index values varied significantly over time (one-way ANOVA, *p* < 0.05). Post hoc comparisons revealed that the value at Y3 (9.568 ± 0.16) was significantly higher than at all other time points, while no significant differences were detected among Y1, Y5, Y8 and Y10. In contrast, the Simpson index remained stable throughout the study period, with no significant differences among any time points (Figure 2b,c; Table A4). Fungal Chao1 richness varied significantly over time (one-way ANOVA, *p* < 0.05) (Figure 2d; Table A4). Post hoc comparisons showed that Y8 (448.33 ± 50.49) had significantly lower richness than all other time points, whereas no significant differences were detected among Y1 (571.51 ± 45.23), Y3 (638.69 ± 134.83), Y5 (574.81 ± 54.41), and Y10 (618.65 ± 164.11). In contrast, fungal Shannon and Simpson remained stable across all sampling years (one-way ANOVA, *p* > 0.05 for both), with no significant differences among any time points (Figure 2e,f; Table A4).

**Figure 3:**
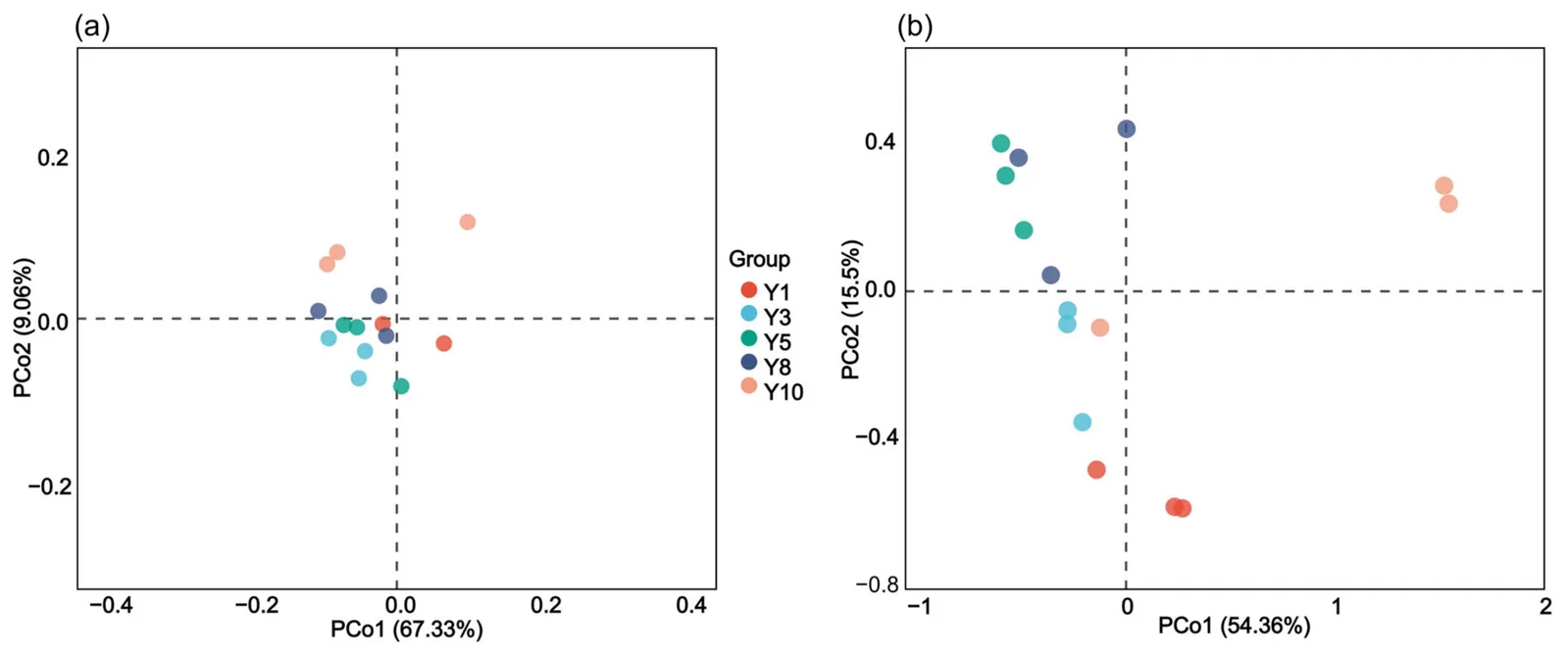
Principal coordinate analysis (PCoA) of soil bacterial (**a**) and fungal (**b**) communities in the rhizosphere of *Paeonia ostii* ‘Feng Dan’ across the continuous cropping chronosequence. Explanations: Same as for Table 1.

Principal coordinate analysis (PCoA) based on Bray–Curtis distances was used to assess bacterial and fungal community dissimilarities across the continuous cropping chronosequence (Figure 3). For bacteria, PCoA1 explained 67.3% of the bacterial variance and PCoA2 explained 9.1%. Along PCoA2, samples from Y10 and most Y8 showed positive scores while Y1, Y3, and Y5 showed negative scores (Figure 3a). PERMANOVA confirmed that cropping duration significantly affected bacterial community composition (Table A5). PERMDISP analysis confirmed that multivariate dispersion did not differ significantly among cropping durations (*F* = 0.36, *p* = 0.834), supporting the validity of the PERMANOVA results. Pairwise comparisons revealed significant differences between Y3 and Y8 (*R*^2^ = 0.27, *p* = 0.001), Y3 and Y10 (*R*^2^ = 0.45, *p* = 0.001), Y5 and Y10 (*R*^2^ = 0.43, *p* = 0.001), and Y8 and Y10 (*R*^2^ = 0.35, *p* = 0.001), whereas no significant differences were detected between Y1 and any other year (all *p* > 0.05) or between Y5 and Y8 (*p* = 0.3).

For fungi, PCoA1 explained 54.4% and PCoA2 explained 15.5% of the variation (Figure 3b). Along PCoA2, Y5, Y8, and most Y10 samples had positive scores, while most Y1 and Y3 samples had negative scores; one Y10 sample clustered near two Y3 samples (Figure 3b). PERMANOVA confirmed that cropping duration significantly affected fungal community composition (Table A5). PERMDISP analysis confirmed that multivariate dispersion did not differ significantly among cropping durations (*F* = 0.64, *p* = 0.644), supporting the validity of the PERMANOVA results. Pairwise comparisons revealed that Y1 differed significantly from Y3 (*R*^2^ = 0.49, *p* < 0.001), Y8 (*R*^2^ = 0.46, *p* < 0.001), and Y10 (*R*^2^ = 0.56, *p* < 0.001). Y3 also differed significantly from Y10 (*R*^2^ = 0.52, *p* < 0.001), and Y8 differed from Y10 (*R*^2^ = 0.31, *p* < 0.001). No significant differences were detected between Y5 and any other year (all p > 0.05) nor between Y3 and Y5 (*p* = 0.1), Y3 and Y8 (*p* = 0.2), or Y5 and Y8 (*p* = 0.1).

### 3.4. Soil Microbiome Assembly in the Rhizosphere of Paeonia ostii ‘Feng Dan’ During Different Cropping Durations

**Figure 4:**
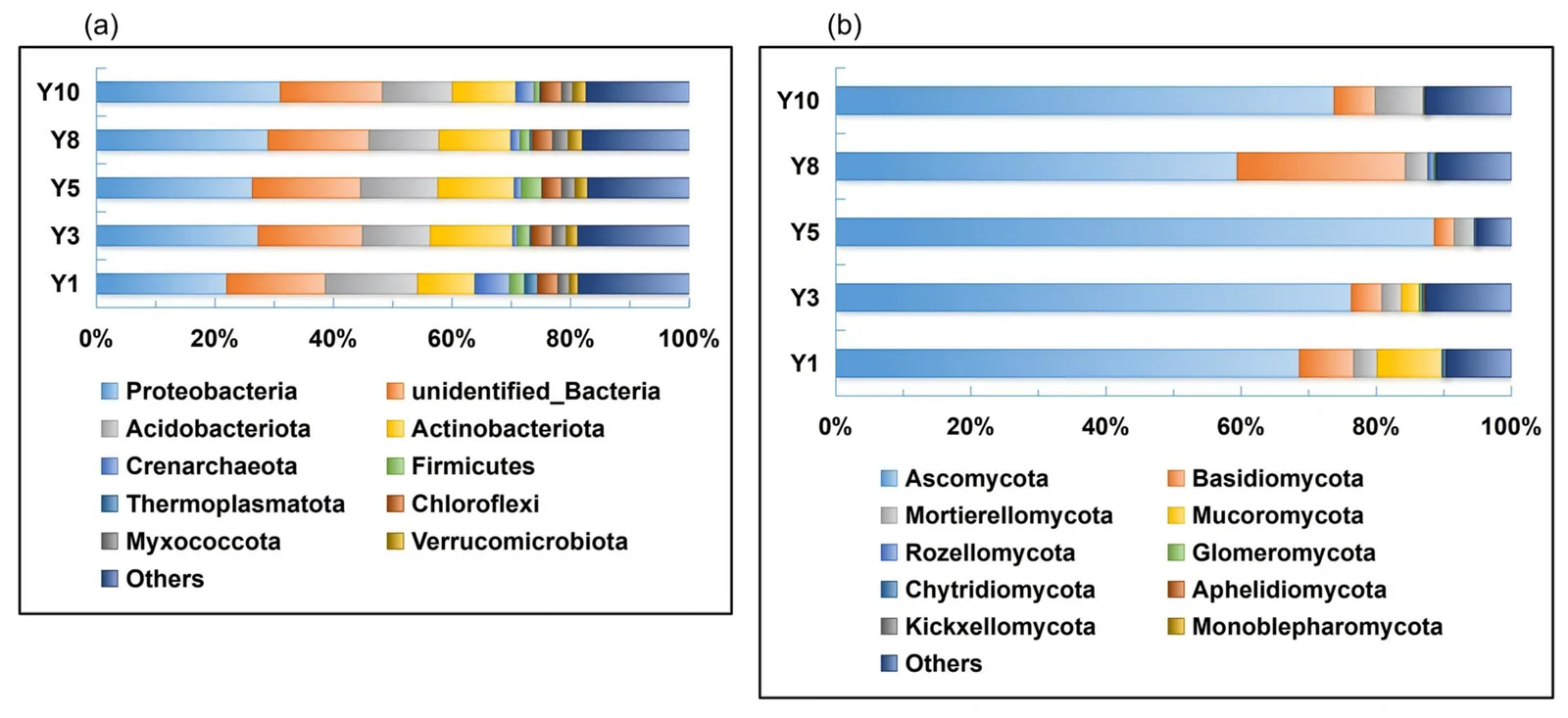
Distribution characteristics of predominant bacterial (**a**) and fungal **(b**) taxa in the rhizosphere soils of *Paeonia ostii* ‘Feng Dan’ across the continuous cropping chronosequence. Explanations: Same as for Table 1.

Across all groups, the dominant bacterial phyla (relative abundance > 1%) included were Proteobacteria (21.9–31%), unclassified bacteria (16.6–18.2%), Acidobacteriota (11.7–15.5%), Actinobacteriota (9.7–13.9%), Crenarchaeota (0.7–5.8%), Firmicutes (0.9–3.4%), Thermoplasmatota (0.09–2.1%), Chloroflexi (3.2–3.4%), Myxococcota (1.9–2.6%), Verrucomicrobiota (1.4–2.3%), Bacteroidota (2.0–2.5%), Gemmatimonadota (1.6–2.8%), and Nitrospirota (1.0–1.9%) (Figure 4a). The highest abundance in Thermoplasmatota (2.2%) and Crenarchaeota (5.8%) appeared in Y1, whereas both phyla remained below 1% in all other groups. Among the dominant phyla, significant differences among cropping years were detected for Proteobacteria and Acidobacteriota (one-way ANOVA, *p* < 0.05). For Proteobacteria, the relative abundance increased significantly in later years: Y8 (0.289 ± 0.01) and Y10 (0.311 ± 0.01) were significantly higher than Y1 (0.219 ± 0.03) (*p* < 0.05), while Y3 (0.273 ± 0.01) and Y5 (0.263 ± 0.02) showed intermediate values that did not differ significantly from either Y1 or the later groups. For Acidobacteriota, the relative abundance declined after Y1: Y3 (0.114 ± 0.01), Y8 (0.118 ± 0.01), and Y10 (0.117 ± 0.01) were significantly lower than Y1 (0.155 ± 0.01) (*p* < 0.05), whereas Y5 (0.130 ± 0.01) was not significantly different from Y1. In contrast, Actinobacteriota and all other phyla—including Chloroflexi, Crenarchaeota, Bacteroidota, Myxococcota, Gemmatimonadota, Firmicutes, Verrucomicrobiota, and Nitrospirota—showed no significant changes over time (*p* > 0.05) (Table A6). For fungi, 11 distinct phyla were detected across all samples (Figure 4b). Ascomycota was the dominant phylum across all treatment groups, accounting for 59.4–88.6% of total sequences, followed by Basidiomycota, Mortierellomycota, and Mucoromycota. One-way ANOVA showed that Ascomycota, Basidiomycota, and Mortierellomycota did not vary significantly along the cultivation chronosequence (all *p* > 0.05; Table A6), although Ascomycota peaked at Y5 (88.6%) and Basidiomycota increased numerically at Y8 (0.249 ± 0.17). In contrast, Mucoromycota varied significantly with cropping duration (*p* < 0.05), being highest at Y1 (0.096 ± 0.01), decreasing sharply at Y3 (0.026 ± 0.02), and not being detected at Y5, Y8, and Y10.

Among the 450 bacterial genera detected across all sampling sites, the most abundant (>1%) were *Sphingomonas* (2.5–4.6%), *RB41* (2.4–5.2%), *MND1* (2.0–2.6%), *Dongia* (1.7–3.2%), and *Steroidobacter* (1.2–1.7%) (Table 2). With increasing cropping duration, *RB41* decreased significantly from 5.2% at Y1 to 2.8% at Y10 (a decrease of 46.2%; *p* < 0.05), while *Lysobacter* increased significantly from 0.5% at Y1 to 1.7% at Y10 (an increase of 240%; *p* < 0.05). No significant changes were observed for the other dominant genera (all *p* > 0.05).

For fungi, 428 genera were identified across all sampling sites. Among the dominant fungal genera, *Fusarium* decreased significantly with cropping duration, and *Monographella* increased from 13.1% at Y1 to 43.0% at Y10 (228% increase; *p* < 0.05). *Torula* was significantly higher in the early stage (Y1–Y3) than in the later stage (Y5–Y10), while *Orbicula* and *Phoma* both peaked at Y5 (*p* < 0.05). The other dominant fungal genera showed no significant differences (all *p* > 0.05; Table 2).

### 3.5. Relationships Between Soil Parameters and Microbial Diversity Indices

Pearson correlation analysis was performed to explore the relationships between soil physicochemical properties and microbial alpha diversity indices (Table 3). For bacterial communities, *Shannon*, *Simpson*, and *Chao*1 indices were positively correlated with soil pH (*r* = 0.577, 0.692, and 0.774, respectively; *p* < 0.05, *p* < 0.01, and *p* < 0.001) and negatively correlated with AN (*r* = −0.543, −0.599, and −0.628, respectively; *p* < 0.05 for all). In addition, bacterial *Chao*1 was negatively correlated with TN (*r* = −0.598, *p* < 0.05) and SOM (*r* = −0.560, *p* < 0.05). Fungal *Shannon* and *Simpson* indices were negatively correlated with TN (*r* = −0.536 and −0.556, respectively; *p* < 0.01) and AK (*r* = −0.563 and −0.564, respectively; *p* < 0.01). In addition, *Shannon* was also negatively correlated with TK (*r* = −0.514, *p* < 0.01) and AN (*r* = −0.521, *p* < 0.01).

**Figure 5:**
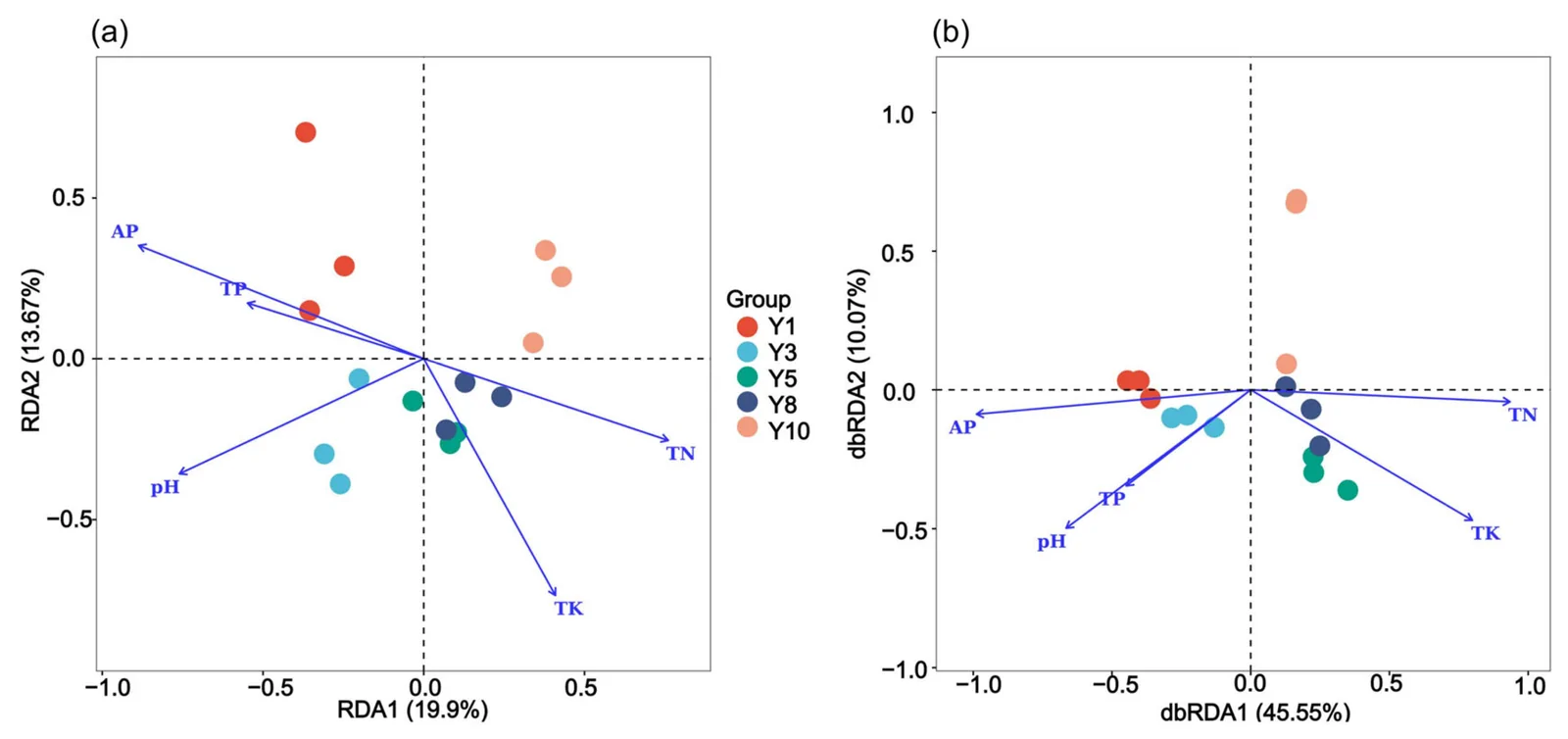
RDA of dominating bacteria (**a**) and fungal (**b**) taxa across the continuous cropping chronosequence. Explanations: Same as for Table 1.

Redundancy analysis (RDA) was performed to examine the relationships between soil properties and bacterial community composition at the genus level (Figure 5a). The five soil variables (TN, TP, TK, AP, and pH) collectively explained 45.55% of the total variation in bacterial community structure, with RDA1 explaining 19.9% and RDA2 explaining 13.67% of the constrained variance. The overall model was highly significant (*F* = 1.918, *p* = 0.003). Monte Carlo permutation tests (999 permutations) revealed that among the five variables, only AP showed a marginally significant correlation with the RDA axes (*r*^2^= 0.1035, *p* = 0.050), while TN (*p* = 0.420), TP (*p* = 0.290), TK (*p* = 0.309), and pH (*p* = 0.143) were not significantly correlated. Along RDA1, the AP and pH vectors pointed toward the negative axis. Y1 and Y3 samples were located on the same (negative) side of these vectors, indicating their association with relatively higher AP and pH conditions during the early stage of continuous cropping. In contrast, Y5, Y8, and Y10 samples projected on the opposite (positive) side, corresponding to lower AP and pH conditions in the later stage.

**Figure 6:**
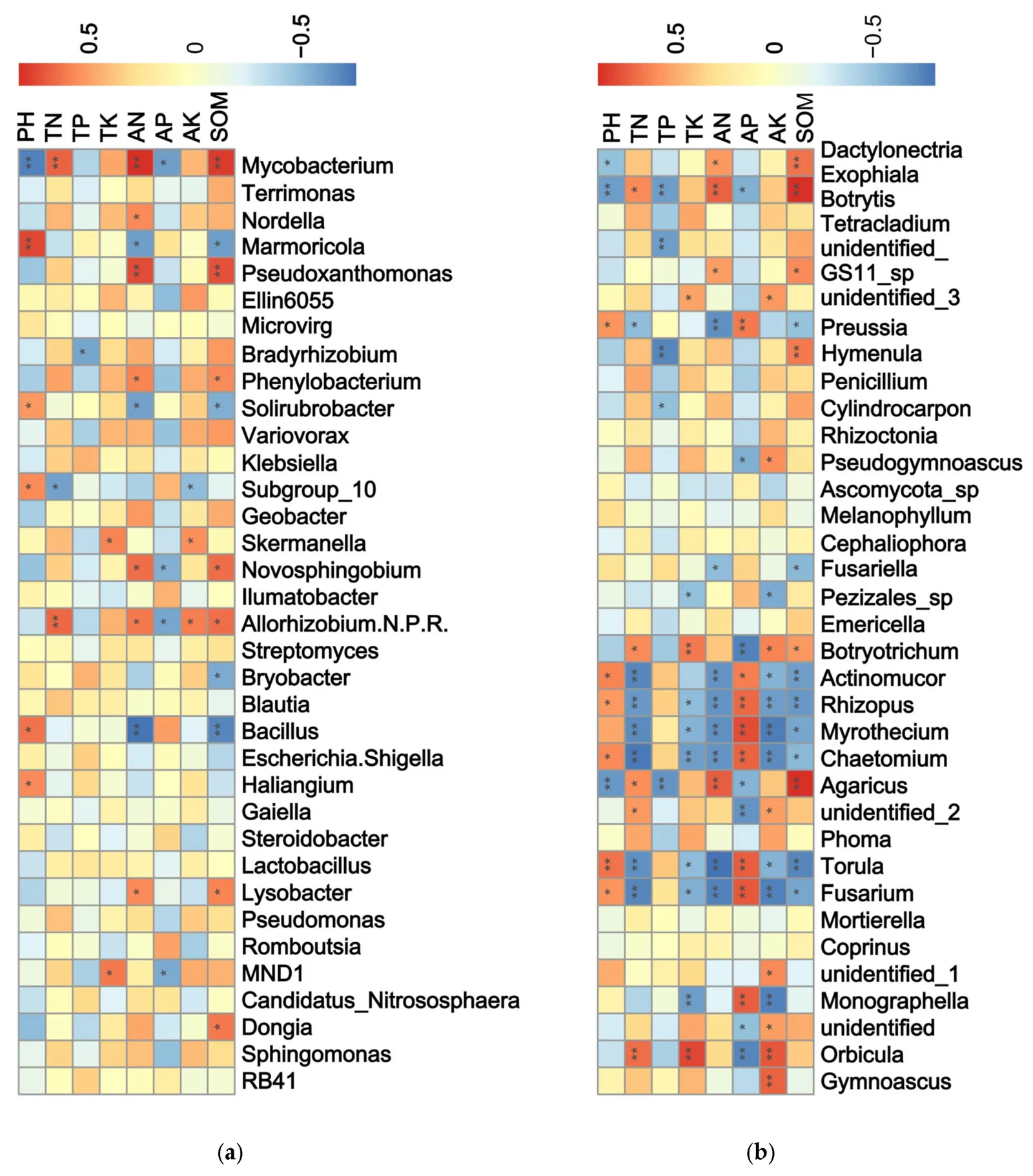
Pearson’s correlation coefficients between soil physicochemical properties and relative abundance of dominant bacterial (**a**) and fungal (**b**) genera across the continuous cropping chronosequence. * *p* < 0.05; ** *p* < 0.01.

Pearson correlation analysis further revealed the significant positive correlations of SOM and AN with *Mycobacterium*, *Pseudoxanthomonas*, *Rhizobium*, *Novosphingobium*, *Lysobacter*, and *Phenylobacterium* (Figure 6a). In contrast, these soil factors were negatively correlated with *Bacillus*, *Marmoricola*, and *Solirubrobacter*. AP was positively correlated with *Bacillus* and *Romboutsia* but negatively correlated with *Mycobacterium*, *Rhizobium*, and *Novosphingobium*. Additionally, pH positively affected *Marmoricola*, *Bacillus*, *Mycobacterium*, and *Solirubrobacter* abundance.

For fungal communities, db-RDA revealed that the five soil variables (TN, TP, TK, AP, and pH) collectively explained 63.17% of the total variation in fungal community composition, with dbRDA1 and dbRDA2 accounting for 45.55% and 10.07% of the constrained variance, respectively (Figure 5b). The overall model was highly significant (*F* = 3.086, *p* = 0.002). Monte Carlo permutation tests (999 permutations) showed that AP (*r*^2^ = 0.783, *p* = 0.001), TK (*r*^2^ = 0.760, *p* = 0.001), TN (*r*^2^ = 0.739, *p* = 0.003) and pH (*r*^2^ = 0.520, *p* = 0.010) were significantly correlated with the db-RDA axes, with AP showing the strongest overall correlation. Along dbRDA1, the AP and pH vectors pointed toward the negative axis. Y1 and Y3 samples were located on the same (negative) side of these vectors, indicating their association with relatively higher AP and pH conditions during the early stage. Conversely, Y5, Y8, and Y10 samples projected on the positive side, corresponding to lower AP and pH conditions in the later stage.

Pearson correlation analysis further revealed that AK and TN were positively correlated with the relative abundances of *Orbicula*, *Botryotrichum*, and *Phoma* but negatively correlated with those of *Myrothecium*, *Fusarium*, *Chaetomium*, *Rhizopus*, and *Torula* (Figure 6b). Conversely, AP and pH exhibited opposite trends, being positively associated with the latter five genera and negatively with the former three.

**Figure A2:**
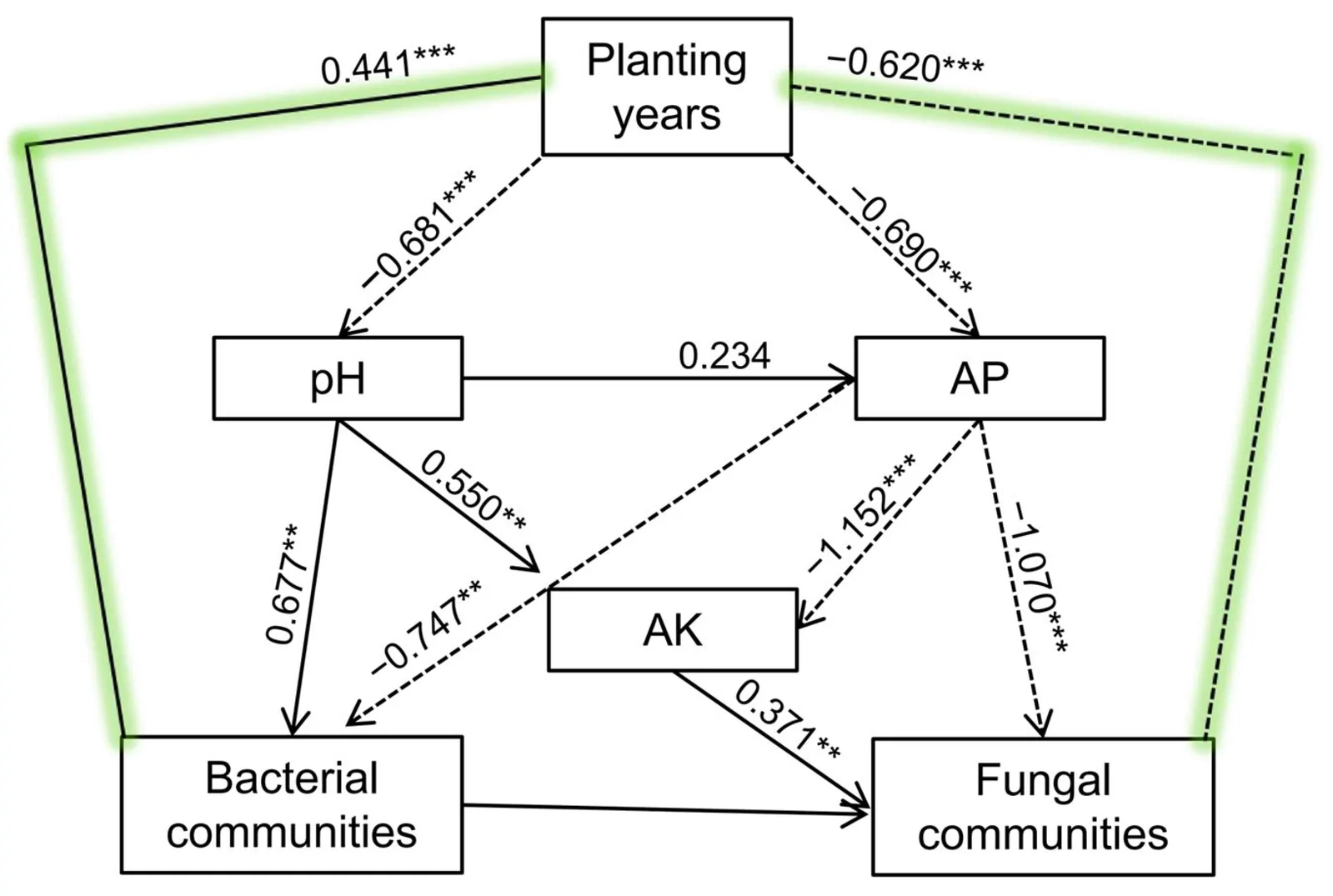
SEM showing the effects of soil properties on microbiome interactions. Numbers on arrows are standardized path coefficients (solid, positive; dashed, negative); model fit: chi-square = 0.051 (df = 2, *p* = 0.975), CFI = 1, RMSEA = 0.000, and GFI = 0.999. ** *p* < 0.01; *** *p* < 0.001.

SEM was used to examine potential relationships among cropping duration, soil properties, and microbial communities (Figure A2). The model explained 65.2% and 91.7% of variance in bacterial and fungal communities, respectively. Cropping duration was associated with bacterial and fungal community composition, and these associations may be at least partially mediated through changes in soil pH and AP. The model also indicated potential negative associations between soil AP and both bacterial and fungal community structures. Given the limited sample size *n* = 15, these exploratory results should be interpreted with caution and require validation in future studies.

### 3.6. Predictive Functional Annotation of Bacteria and Fungi

**Figure 7:**
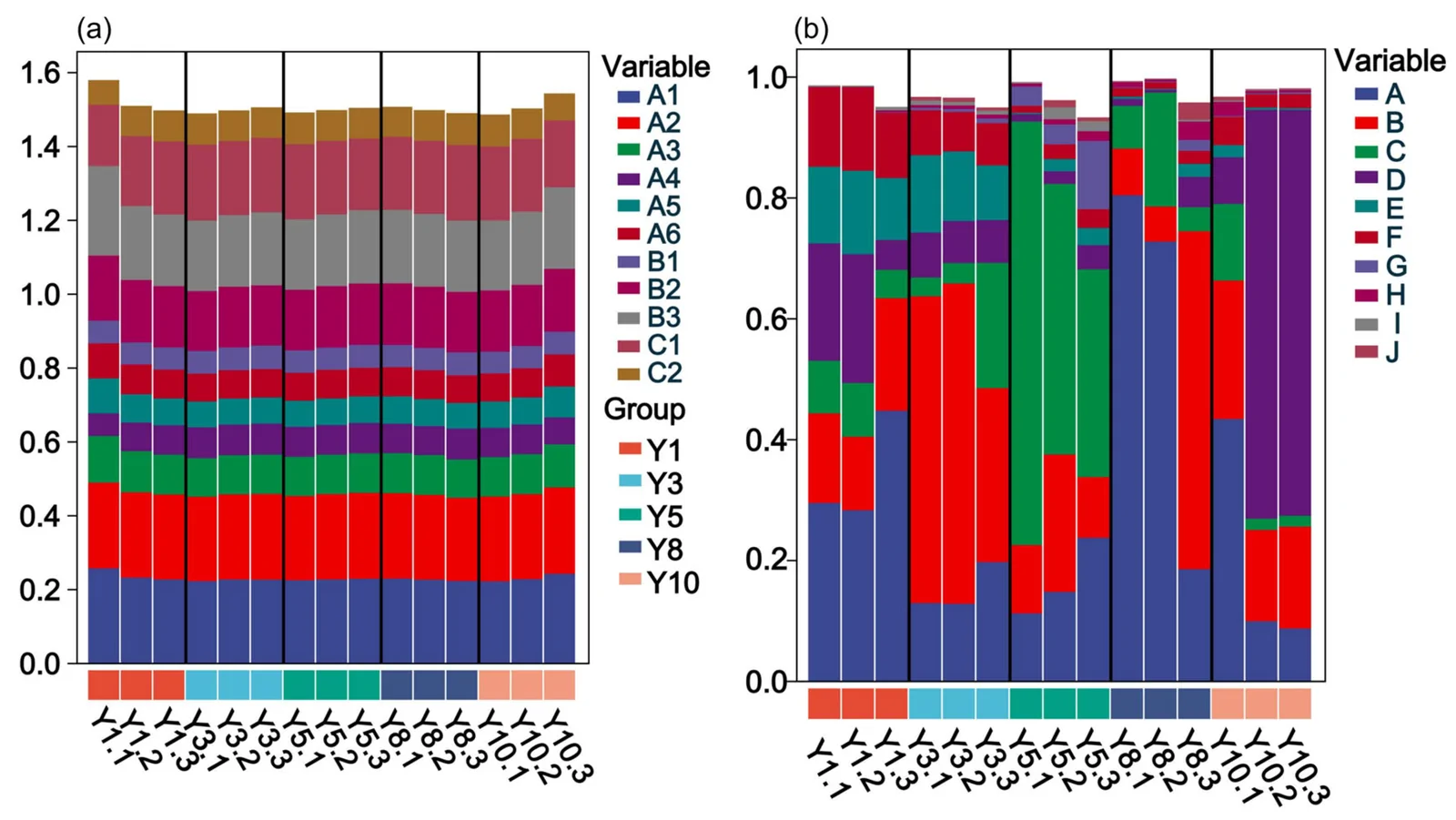
Effects of continuous cropping duration on the rhizosphere microbiome. Relative abundance of (**a**) KEGG secondary metabolic pathways (level II) in bacteria, and (**b**) fungal functional guilds (FUNGuild), across different cropping durations. See Table A7 for full names of pathways and guilds.

Predicted functional classification of bacterial communities was performed by comparing inferred functional gene profiles with the KEGG Orthology (KO) database. Six major predicted biological metabolic functions were observed at the first functional level-1: cellular processes, environmental information processing, genetic information processing, human diseases, metabolism, and organismal systems. Among these, metabolism, genetic information processing, and environmental information processing were the primary first-level functions. At KEGG level-2, 44 predicted secondary functional categories were identified, of which 11 had predicted relative abundances greater than 1% (Figure 7a); only replication and repair, membrane transport, and energy metabolism showed significant changes with cropping duration (*p* < 0.05); the remaining eight categories remained stable (*p* > 0.05). Carbohydrate metabolism and amino acid metabolism were the most abundant predicted categories, followed by translation, membrane transport, replication and repair, and energy metabolism. All other secondary categories had predicted relative abundances below 10%.

Predicted fungal trophic modes were classified using FUNGuild (Figure 7b). The dominant predicted nutritional guilds across all samples were saprotrophs (25.84–67.33%), pathotrophs (2.37–48.09%), and pathotrophs-Saprotrophs (3.22–24.56%). ANOVA identified two guilds with significant abundance shifts (>1%) over time: Undefined_Saprotroph (primarily associated with *Gymnoascus* and *Agaricaceae*) and Plant_Pathogen-Soil_Saprotroph-Wood_Saprotroph (predominantly linked to *Fusarium*). The confidence of FUNGuild assignments varied among taxa: an amount of 7.1% (113 OTUs) were classified as “highly probable”, 27.6% (437 OTUs) as “probable”, 8.6% (137 OTUs) as “possible”, and 56.6% (897 OTUs) remained unassigned.

**Figure 8:**
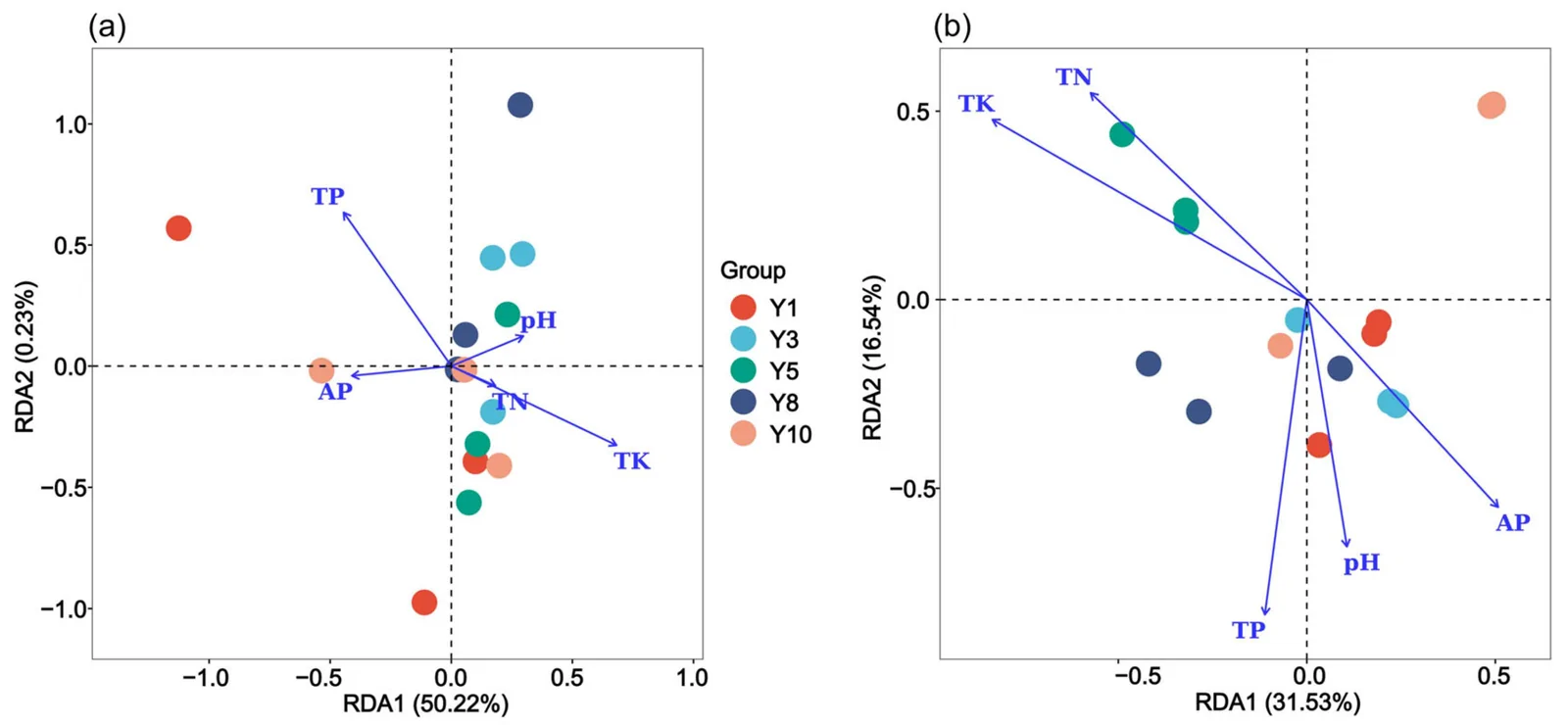
RDA of the relationships between soil physicochemical properties and (**a**) bacterial functional genes and (**b**) the relative abundance of fungal guilds. Explanations: Same as for Table 1.

RDA was performed to examine the relationships between soil properties and bacterial functional composition (Figure 8a). The five soil variables collectively explained 50.66% of the variance; however, the overall model was not statistically significant (*F* = 1.85, *p* = 0.156). RDA1 separated the early stage (Y1) from the later cropping years (Y3–Y10). Envfit analysis revealed no significant correlations between any measured soil variable and the RDA axes (all *p* > 0.05). Thus, the measured soil properties did not significantly explain the observed variation in bacterial functional composition.

RDA of fungal functional guilds revealed that the five edaphic variables collectively explained 67.18% of the variance, with the overall model being highly significant (*F* = 3.68, *p* = 0.001) (Figure 8b). The first two constrained axes accounted for 31.53% and 16.54% of the explained variance, respectively. RDA1 clearly discriminated Y1, Y3, and Y10 (negative side) from Y5 and Y8 (positive side). Envfit analysis further identified TN (*r*^2^ = 0.5225, *p* = 0.006), TP (*r*^2^ = 0.5350, *p* = 0.014), TK (*r*^2^ = 0.7485, *p* = 0.001), and AP (*r*^2^ = 0.4657, *p* = 0.027) as significantly associated with the ordination axes. Collectively, these findings suggest that TN, TK, and AP are key edaphic factors shaping fungal functional composition along the cropping chronosequence.

## 4. Discussion

### 4.1. Soil Chemical Changes and Edaphic Drivers of Microbial Community Differentiation During Long-Term Monoculture of Paeonia ostii ‘Feng Dan’

In this study, continuous cropping of *Paeonia ostii* ‘Feng Dan’ for up to 10 years induced a striking decoupling in soil chemistry: while SOM, TN, and AN accumulated from Y5 onward, AP plummeted by ~50% after Y3 and remained depleted thereafter. The SOM and TN buildup likely reflects sustained organic residue input [52,53,54], whereas the AP decline suggests transformation of labile P into microbial biomass or stable mineral phases through microbial immobilization and physicochemical sorption [55,56]. This decoupling challenges the conventional assumption that organic matter accumulation automatically improves phosphorus availability [57]. db-RDA identified AP as the strongest predictor associated with bacterial community variation, highlighting the critical role of phosphorus availability in structuring bacterial assemblages in this agroecosystem. For fungi communities, AP, TK, TN, and pH collectively showed significant correlations with community composition. The higher explanatory power of environmental variables for fungi (63.17%) than for bacteria (45.55%) suggests that fungi are more responsive to phosphorus depletion in this alkaline system [58,59], potentially reflecting their distinct ecological strategies under alkaline pH conditions where phosphorus is the primary limiting resource.

Notably, although the average content of AP declined significantly with prolonged monoculture, RDA indicated that AP remained a key factor associated with bacterial community differentiation from Y5 toY10. This reflects that RDA captures relative gradients among samples rather than absolute nutrient status [60]: even under overall AP depletion, the remaining inter-sample variability in AP was sufficient to structure the community. The sharp transition from high to low AP between Y3 and Y5 may therefore constitute a strong environmental filter, selecting for taxa with low-phosphorus adaptation strategies (e.g., *Geobacter* and *Massilia*) [61]. Genus-specific correlations supported this: *Bacillus* was positively associated with AP and pH, and its relative abundance decreased from 0.44% at Y1 to 0.24% at Y10, suggesting adaptation to alkaline, phosphorus-rich conditions and a decline under phosphorus depletion [62,63]. Conversely, *Rhizobium* and *Novosphingobium*—key taxa involved in nitrogen fixation [64,65] and phosphate solubilization [66], respectively—were negatively correlated with AP and pH, and their relative abundances increased substantially over time (*Rhizobium*: from 0.18% to 0.59%; *Novosphingobium*: from 0.17% to 0.85%). These opposing trends indicate that continuous cropping drove a functional succession from phosphorus-rich-adapted taxa to phosphorus-limited-adapted taxa, with the enriched *Rhizobium* and *Novosphingobium* potentially contributing to the maintenance of nitrogen and phosphorus cycling functions under long-term cropping stress.

The Y3–Y5 interval marked a critical transition window, during which the RDA ordination shifted from negative to positive AP associations. Over this period, AP declined by nearly half, while TN, AN, SOM, and AK began to accumulate. This interval is proposed to trigger a regime shift in microbial community assembly, ultimately contributing to replanting syndrome [67]. From a management perspective, these findings highlight that AP [68], not TP or other macronutrients, is the most critical limiting factor in long-term *Paeonia ostii* ‘Feng Dan’ monoculture. Moreover, the decoupling of AP from SOM/TN accumulation indicates that organic matter buildup does not automatically replenish AP, emphasizing the need for targeted phosphorus management such as phosphate-solubilizing microorganisms or periodic P fertilization [63].

### 4.2. Divergent Successional Patterns of Bacterial and Fungal Communities Under Continuous Cropping

In this study, bacterial and fungal communities exhibited distinct successional patterns under prolonged monoculture. While bacterial richness (*Chao1*) showed an overall decline with minor fluctuations along the chronosequence, the concurrent stability of *Simpson* evenness suggests that the observed species loss did not translate into a major restructuring of community dominance. This decoupling points to the selective elimination of rare taxa rather than a community-wide collapse [69,70,71]. In contrast to the bacterial successional pattern, fungal communities exhibited a distinct response. Fungal richness remained higher than at Y1 at most cropping stages but declined markedly at Y8. The stability of fungal *Shannon* and *Simpson* indices throughout the study period suggests that hyphal networks may buffer fungal communities against richness fluctuations by maintaining functional redundancy [72].

Correlation analyses further revealed that bacterial alpha diversity was most strongly associated with pH (positive) and AN (negative), suggesting that shifts in soil pH and nitrogen accumulation under continuous cropping may contribute to bacterial richness decline. This aligns with previous findings that soil pH is a strong predictor of bacterial community composition [73,74] and that bacterial diversity often declines under elevated nitrogen availability [75]. Notably, while fungal richness was unrelated to any measured edaphic factor, fungal evenness exhibited associations with soil nutrients. This differential sensitivity suggests that fungal community evenness, rather than richness, may respond to nutrient accumulation under long-term monoculture, further highlighting the divergent responses of bacterial and fungal communities to soil properties [76].

The decrease in bacterial richness suggests that long-term monoculture creates a selective environment—characterized by altered pH, root exudates, and pesticide residues—that eliminates sensitive taxa [77,78]. In contrast, continuous input of root debris and organic matter provides new niches for saprophytic fungi. These divergent responses reflect fundamental ecological principles: bacterial communities, with faster growth rates and narrower niche preferences, are more vulnerable to selective pressures and changes in pH, nitrogen, and organic matter [79,80], whereas, fungal communities, with broader enzymatic capabilities and extensive hyphal networks, are more resilient and can colonize new niches, maintaining diversity even under nutrient fluctuations [81,82].

### 4.3. Succession of Fungal Pathogens and the Replanting Syndrome

In this study, *Fusarium*, *Monographella*, and *Mortierella* persisted as the core dominant genera across all stand ages. The first two taxa are recognized plant pathogens [83,84]. Their divergent temporal dynamics suggest a distinct pathogen succession: *Fusarium* dominated the early stage but declined thereafter, whereas *Monographella* exhibited a late-stage surge, becoming the dominant pathogen after 8–10 years of continuous cropping. The delayed enrichment in *Monographella* is particularly significant, as this genus contains species known to cause leaf and stem blight in various crop [85]. A mid-phase outbreak of *Phoma*—another pathogenic genus [86]—was also observed, indicating a temporal shift in pathogen dominance. In contrast, *Torula*, often considered a saprophyte or weak opportunist [87], declined over time, suggesting that long-term monoculture favors pathogenic over saprophytic taxa. The late-stage emergence of *Monographella,* coupled with the decline in other pathogens, implies a narrowing of the pathogen community toward a single dominant taxon, which may exacerbate replanting syndrome in the later years of *Paeonia ostii* continuous cropping.

This succession is fundamentally linked to the long-term changes in soil chemistry. The positive correlations of *Fusarium* abundance with AP and pH suggest that the progressive depletion in AP and the modest decline within the persistently alkaline range may have contributed to its suppression after the initial years. In contrast, *Monographella* abundance was positively associated with TN and SOM but negatively correlated with pH, implying that the accumulation of organic matter and nitrogen from Y5 onward may favor its growth, while the concurrent pH decline may have further facilitated rather than constrained its growth [88]. At the community level, RDA revealed that TN, TK, AP, and pH collectively shaped the fungal community structure in the later years (Y5, Y8, and Y10). These edaphic factors likely facilitated the enrichment of pathogenic genera, including *Monographella* and *Phoma*, during this phase [88,89].

The stark contrast in fungal community composition between the early (Y1, Y3) and later (Y5–Y10) years, paralleling the marked shifts in edaphic properties, underscores a fundamental nutritional transition in the soil ecosystem. This pattern is consistent with previous studies reporting increased levels of certain nutrients, including TN and AK, following long-term continuous cropping [77,90]. Notably, AK exhibited a strong negative correlation with early-stage pathogens such as *Fusarium* and *Myrothecium*, suggesting that potassium depletion over the cropping sequence may have eroded the soil’s natural suppressiveness against these taxa [90,91]. Collectively, these findings indicate that the progressive alteration of soil nutrient stoichiometry not only restructures the fungal community but also selectively favors the proliferation of pathogenic lineages [88,92].

An apparent paradox emerged: although *Fusarium* abundance was positively correlated with AP, the overall soil-borne disease risk escalated as AP declined along the cropping chronosequence, culminating in the *Monographella* dominance at Y10. This discrepancy indicates that pathogen enrichment is not driven by a single nutritional factor but reflects species-specific responses to multiple edaphic stressors. Specifically, while *Fusarium* thrives under phosphorus-rich conditions [93], persistent alkaline pH condition [94] and potassium depletion [95] characteristic of this system may have created a niche conducive to opportunistic pathogens such as *Monographella*. Additionally, continuous cropping-induced alterations in root exudate composition may provide alternative carbon and nutrient sources [96,97], decoupling pathogen proliferation from soil phosphorus status. Thus, the net expansion of pathogenic communities persisted despite AP depletion. Importantly, this expansion was not counteracted by the resident biocontrol fungus *Mortierella* [98], which remained dominant throughout but did not prevent the late-stage surge of *Monographella*. Its biocontrol functions are primarily mediated through rhizosphere microbiome modulation rather than direct pathogen inhibition [99], and such indirect suppression may be insufficient once pathogen pressure escalates—particularly when pathogens expand through mechanisms that circumvent resource competition [96,97]. It is noteworthy, however, that RDA identified AP as a significant structuring force for the overall microbial community, implying that phosphorus availability primarily governs community assembly rather than the absolute abundance of specific pathogens [100].

Taken together, our results suggest a temporal succession of fungal pathogens under continuous tree peony cropping: *Fusarium* predominates the early phase, *Phoma* peaks in the middle phase, and *Monographella* becomes the dominant taxon in the late phase (Y10). The replanting syndrome of *Paeonia ostii* may therefore not merely result from pathogen accumulation but may also reflect a shifting pathogen complex, in which the dominant causal agent changes with cropping duration. Accordingly, management strategies should be stage-specific: control *Fusarium* in early years (e.g., seed treatment or soil disinfestation) [101,102], target *Phoma* at Y5, and focus on *Monographella* in later years by reducing excess nitrogen and carbon inputs [54], maintaining near-neutral pH [103,104], and enhancing antagonistic bacteria such as phosphate-solubilizing *Bacillus* strains associated with AP [105].

### 4.4. Functional Shifts in the Soil Microbiome Under Continuous Cropping

At the broad functional level, carbohydrate and amino acid metabolism were predicted as the dominant pathways across the chronosequence, consistent with their central role in soil microbial metabolism [106]. Membrane transport and secondary metabolism were also predicted as relatively abundant categories. These inferred patterns broadly tracked the observed taxonomic shifts, with predicted declines in energy metabolism and replication functions in later years alongside a relative increase in membrane transport functions suggesting an adaptive strategy of microbes for coping with these stresses [106]. However, as these are computationally inferred rather than experimentally determined, they are presented here as a putative framework requiring further functional validation.

Predicted functional profiles suggested a differential sensitivity of bacterial and fungal putative functions to edaphic changes along the chronosequence. For bacteria, soil properties explained 50.66% of the inferred functional variance, yet the RDA model was not significant (*F* = 1.85, *p* = 0.156), suggesting that the predicted bacterial functional composition was largely decoupled from the measured soil chemical gradients. In contrast, fungal functional guilds showed a stronger predicted association with soil nutrients (67.18% explained variance, *F* = 3.68, *p* = 0.001), with TN, TP, TK, and AP significantly correlated with the ordination axes. This inferred fungal pattern aligns with the observed nutrient-driven succession of fungal pathogens (e.g., *Monographella*) [88], though it should be noted that these are computationally predicted rather than experimentally validated functions. Consequently, the functional shifts described here serve primarily as a putative framework complementary to the taxonomic patterns, and their biological relevance awaits metagenomic or transcriptomic confirmation.

In summary, the predicted functional profiles partially paralleled the observed taxonomic succession: fungal inferred guilds showed significant associations with soil nutrients, whereas bacterial predicted functions were largely decoupled from the measured edaphic gradients. These patterns highlight the multifactorial nature of microbial responses to continuous cropping, though they should be interpreted cautiously because the functional profiles were computationally inferred rather than experimentally measured. Whether such putative functional shifts contribute to replant disease etiology in *Paeonia ostii* ‘Feng Dan’ remains to be validated through metatranscriptomics, metaproteomics, or targeted enzyme assays.

## 5. Conclusions

In this study, we demonstrate that continuous cropping of *Paeonia ostii* ‘Feng Dan’ induces predictable successional dynamics in soil microbiomes. Bacterial richness showed an overall decline with minor fluctuations along the chronosequence, whereas fungal richness displayed a non-monotonic pattern, remaining above the initial level at most stages but showing a pronounced depression at Y8. Fungal communities underwent a directional turnover from *Fusarium* dominance in early years to *Phoma* at the middle stage and ultimately to *Monographella* by year 10. This temporal replacement indicates that replanting syndrome arises from a stage-specific pathogen complex rather than the accumulation of a single pathogen—a shift closely linked to the decoupling of phosphorus availability from organic matter accumulation under alkaline soil conditions. At the functional level, predicted bacterial profiles shifted toward stress-adaptive processes, indicating a metabolic trade-off between growth and maintenance under long-term monoculture stress. Collectively, these findings demonstrate that phosphorus availability—rather than bulk organic matter accumulation—is the primary factor shaping microbial succession and pathogen dynamics in this alkaline agroecosystem. Management should therefore adopt stage-specific interventions targeting each dominant pathogen, coupled with phosphorus optimization, to mitigate replant disease.

## Figures and Tables

**Table 1:** Soil physicochemical properties across the continuous cropping chronosequence.

Soil Factor	Y1	Y3	Y5	Y8	Y10	One-Way ANOVA
pH	8.38 ± 0.06 ab	8.48 ± 0.04b	8.31 ± 0.00 a	8.30 ± 0.03 a	8.25 ± 0.06 a	F = 10.12, *p* = 0.002
TN (g kg^−1^)	0.75 ± 0.02 b	0.73 ± 0.02 b	1.03 ± 0.08 a	0.99 ± 0.08 a	0.95 ± 0.01 a	F = 21.69, *p* = 0.000
TP (g kg^−1^)	1.02 ± 0.08 b	0.95 ± 0.02 ab	0.87 ± 0.04 a	1.02 ± 0.06 b	0.84 ± 0.00 a	F = 9.03, *p* = 0.002
TK (g kg^−1^)	20.06 ± 0.48 a	20.39 ± 0.34 a	21.50 ± 0.241 b	20.87 ± 0.26 ab	20.61 ± 0.17 a	F = 8.95, *p* = 0.002
AN (mg kg^−1^)	54.21 ± 3.62 b	43.03 ± 4.35 a	65.73 ± 1.48 c	75.83 ± 4.14 d	78.71 ± 4.29 d	F = 48.05, *p* = 0.000
AP (mg kg^−1^)	21.07 ± 1.40 b	19.40 ± 1.25 b	9.78 ± 0.09 a	9.15 ± 0.91 a	10.08 ± 0.58 a	F = 108.29, *p* = 0.000
AK (g kg^−1^)	0.08 ± 0.00 a	0.12 ± 0.01 a	0.15 ± 0.01 b	0.16 ± 0.02 b	0.13 ± 0.00 ab	F = 26.48, *p* = 0.000
SOM (g kg^−1^)	11.12 ± 0.57 b	10.34 ± 0.28 b	15.12 ± 0.29 c	15.28 ± 1.10 c	19.87 ± 0.52 a	F = 111.18, *p* = 0.000

Y1, Y3, Y5, Y8, and Y10 indicate continuous cropping for 1, 3, 5, 8, and 10 years, respectively. Data are presented as mean ± standard error n = 3. Different lowercase letters within the same row indicate significant differences among cropping durations (*p* < 0.05, one-way ANOVA followed by LSD post hoc test). Units are indicated in parentheses in the table header. Abbreviations are defined in the Section 2.

**Table 2:** Relative abundance (%) of dominant bacterial and fungal genera across the continuous cropping chronosequence.

Bacteria Genus	Y1	Y3	Y5	Y8	Y10
*Sphingomonas*	2.5 ± 1.00 a	4.0 ± 0.00 a	3.5 ± 0.00 a	4.6 ± 0.00 a	4.2 ± 0.00 a
*RB41*	5.2 ± 1.00 a	2.4 ± 0.00 ab	3.2 ± 0.00 ab	3.3 ± 1.00 ab	2.8 ± 1.00 b
*MND1*	2.1 ± 0.00 a	2.0 ± 0.00 a	2.6 ± 0.00 a	2.5 ± 0.00 a	2.3 ± 0.00 a
*Dongia*	1.7 ± 0.00 a	1.8 ± 0.00 a	2.0 ± 0.00 a	1.9 ± 0.00 a	3.2 ± 0.00 a
*Steroidobacter*	1.7 ± 0.00 a	1.4 ± 0.00 a	1.3 ± 0.00 a	1.2 ± 0.00 a	1.5 ± 0.00 a
*Candidatus_Nitrososphaera*	1.4 ± 1.00 a	0.2 ± 0.00 a	0.2 ± 0.00 a	0.3 ± 0.00 a	0.5 ± 1.00 a
*Lysobacter*	0.5 ± 0.00 b	0.3 ± 0.00 b	0.3 ± 0.00 b	0.5 ± 0.00 ab	1.7 ± 0.00 a
*Romboutsia*	0.2 ± 0.00 a	0.0 ± 0.00 a	0.9 ± 0.00 a	0.0 ± 0.00 a	0.2 ± 0.00 a
*Pseudomonas*	0.0 ± 0.00 a	0.7 ± 0.00 a	0.9 ± 0.00 a	0.5 ± 0.00 a	0.6 ± 0.00 a
Fungal Genus	Y1	Y3	Y5	Y8	Y10
*Fusarium*	12.5 ± 5.00 a	6.9 ± 4.00 a	2.2 ± 2.00 b	1.6 ± 3.00 b	3.0 ± 6.00 ab
*Monographella*	13.1 ± 6.00 ab	5.5 ± 0.90 b	0.9 ± 1.00 a	1.0 ± 4.00 ab	43.0 ± 3.00 ab
*Mortierella*	3.4 ± 0.00 a	2.8 ± 1.00 a	2.9 ± 0.00 a	3.2 ± 1.00 a	7.1 ± 3.00 a
*Torula*	11.8 ± 1.00 a	11.0 ± 1.00 a	1.7 ± 1.00 b	0.8 ± 1.00 b	0.7 ± 1.00 b
*Orbicula*	0.2 ± 0.00 a	6.5 ± 4.00 ab	48.9 ± 8.00 b	9.0 ± 4.00 ab	4.4 ± 3.00 ab
*Phoma*	0.0 ± 0.00 a	0.5 ± 0.00 b	5.9 ± 5.00 c	0.7 ± 0.00 b	0.2 ± 0.00 b
*Gymnoascus*	0.0 ± 0.00 a	0.1 ± 0.00 a	0.4 ± 0.00 a	23.6 ± 10.00 a	0.0 ± 0.00 a
*Coprinus*	7.6 ± 6.00 a	0.0 ± 0.00 a	0.0 ± 0.00 a	0.0 ± 0.00 a	0.0 ± 0.00 a

Data are presented as mean ± standard error (*n* = 3). Different lowercase letters within the same row indicate significant differences among cropping durations (*p* < 0.05). Only genera with relative abundance >1% in at least one treatment are shown. Statistical methods are described in the Section 2.

**Table 3:** Pearson’s correlation coefficients between soil physicochemical properties and alpha diversity indices of bacterial and fungal communities.

Group	Index	pH	TN	TP	TK	AN	AP	AK	SOM
Bacteria	*Chao* 1	0.77 ***	−0.60 *	0.30	−0.04	−0.63 *	0.21	−0.01	−0.56 *
	*Shannon*	0.58 *	−0.25	0.09	0.15	−0.54 *	0.16	0.24	−0.5
	*Simpson*	0.69 **	−0.46	0.23	−0.09	−0.60 *	0.11	0.04	−0.47
Fungi	*Chao* 1	0.18	−0.20	−0.30	−0.08	−0.31	0.32	−0.17	−0.10
	*Shannon*	0.41	−0.54 **	0.08	−0.51 **	−0.52 **	0.33	−0.56 **	−0.28
	*Simpson*	0.32	−0.56 **	0.11	−0.46	−0.48	0.27	−0.56 **	−0.24

* Values are Pearson’s correlation coefficients (r). Significance levels: * *p* < 0.05, ** *p* < 0.01, *** *p* < 0.001. Abbreviations are defined in the Section 2.

## Data Availability

The dataset is publicly available in the Zenodo repository (DOI: 10.5281/zenodo.17112101).

## Abbreviations

The following abbreviations are used in this manuscript:

TN	Total nitrogen
TP	Total phosphorus
TK	Total potassium
AN	Available nitrogen
AP	Available phosphorus
AK	Available potassium
SOM	Soil organic matter
CFI	Comparative fit i
GFI	Goodness of fit
RMSEA	Root square mean error of approximation
RDA	Redundancy analysis

## Appendix A

**Table A1:** Quality control parameters for soil chemical analyses.

Parameter	Method	LOD	LOQ	Accuracy (Relative Error)	Recovery (%)
Total nitrogen (TN)	NY/T 1121.24-2012	0.01 g/kg	0.04 g/kg	≤±10%	85–115
Total phosphorus (TP)	LY/T 1232-2015	10 mg/kg	40mg/kg	≤±10–±15%	80–120
Total potassium (TK)	LY/T 1234-2015	20 mg/kg	80 mg/kg	≤±10–±15%	85–115
Available phosphorus (AP)	NY/T 1121.7-2014	0.5 mg/kg	2.0 mg/kg	≤±15%	80–120
Available potassium (AK)	NY/T 889-2004	5 mg/kg	20 mg/kg	≤±15%	80–120
Available nitrogen (AN)	LY/T 1228-2015	5 mg/kg	20 mg/kg	≤±15%	75–120
Soil organic matter (SOM)	NY/T 1121.6-2006	0.3 g/kg	1.0 g/kg	≤±10%	85–110

**Table A2:** Equipment and instruments used for soil chemical analyses.

Parameter	Instrument/Equipment	Model	Manufacturer	Country of Origin
pH	pH meter	PHS-3C	Leici (Shanghai INESA)	Shanghai, China
TN	Continuous flow analyzer	AutoAnalyzer III	SEAL Analytical	Norderstedt, Germany
TP	UV–Vis spectrophotometer	UV-1800	Shimadzu	Kyoto, Japan
TK	Flame photometer	FP640	Leici (Shanghai INESA)	Shanghai, China
AP	UV–Vis spectrophotometer	UV-1800	Shimadzu	Kyoto, Japan
AK	Flame photometer	FP640	Leici (Shanghai INESA)	Shanghai, China
AN	Micro-diffusion apparatus	-	-	-
SOM	Titration apparatus	-	-	-

**Table A3:** Statistical analysis of the OTUs with 97% threshold.

	Soil Bacteria					Soil Fungi		
Sample Names	Qualified Seqs	Effective Tags	Aligned Rate (%)	OTUs Number	Qualified Seqs	Effective Tags	OTUs Number	Aligned Rate (%)
Y1.1	53,196	39,966	72.27	1895	82,612	66,079	502	79.87
Y1.2	51,435	34,357	73.64		80,239	63,004		77.52
Y1.3	52,101	38,589	77.22		83,179	63,903		75.6
Y3.1	67,905	50,988	74.7	2043	78,693	62,368	565	78.62
Y3.2	58,400	45,509	76.28		51,887	44,861		85.56
Y3.3	65,446	49,971	72.35		84,773	69,898		81.89
Y5.1	50,981	39,568	71.84	1922	80,506	62,923	513	77.83
Y5.2	53,644	39,881	77.16		79,163	61,112		75.83
Y5.3	54,218	40,217	76.5		83,592	64,898		77.16
Y8.1	56,555	44,093	75.73	1891	82,261	65,646	396	79.42
Y8.2	62,103	48,459	72.33		77,118	61,350		79.14
Y8.3	65,720	50,230	64.91		90,908	69,342		75.8
Y10.1	55,164	40,878	73.26	1803	83,592	68,170	546	81.13
Y10.2	52,360	34,830	74.37		80,678	63,662		77.94
Y10.3	55,905	42,040	65.25		84,594	65,058		76.44

**Table A4:** Soil microbial α-diversity index of *Paeonia ostii* ‘Feng Dan’ rhizospheric soil samples.

**Group**	***Shannon***	***Simpson***	***Chao*** **1**
Bacteria			
Y1	9.205 ± 0.31 a	0.995 ± 0.00 a	2347.75 ± 567.20 a
Y3	9.568 ± 0.16 b	0.997 ± 0.00 a	2340.69 ± 198.25 ab
Y5	9.360 ± 0.06 a	0.996 ± 0.00 a	2095.90 ± 40.13 c
Y8	9.335 ± 0.02 a	0.996 ± 0.00 a	2140.78 ± 133.79 b
Y10	9.165 ± 0.06 a	0.995 ± 0.00 a	1947.70 ± 87.49 d
**Fungi**	***Shannon***	***Simpson***	***Chao*1**
Y1	5.14 ± 0.26 a	0.93 ± 0.004 a	571.51 ± 45.23 a
Y3	5.30 ± 0.70 a	0.88 ± 0.058 a	638.69 ± 134.83 a
Y5	3.91 ± 1.20 a	0.72 ± 0.191 a	574.81 ± 54.41 a
Y8	3.37 ± 1.96 a	0.65 ± 0.255 a	448.33 ± 50.49 b
Y10	4.25 ± 1.68 a	0.72 ± 0.216 a	618.65 ± 164.11 a

The value is mean ± standard error (*n* = 3); different lowercase letters in the same line indicate significant difference between treatments (*p* < 0.05).

**Table A5:** Adonis analysis of bacterial and fungal based on Bray–Curtis.

	Permutational MANOVA		Nonparametric MANOVA	
	R^2^	Pr (>F)	R^2^	Pr (>F)
Bacteria				
Y1–Y3	0.37	0.1	0.63	0.1
Y1–Y5	0.41	0.1	0.59	0.1
Y1–Y8	0.38	0.1	0.62	0.1
Y1–Y10	0.46	0.1	0.54	0.1
Y3–Y5	0.31	0.1	0.69	0.1
Y3–Y8	0.27	0.001	0.73	0.001
Y3–Y10	0.45	0.001	0.55	0.001
Y5–Y8	0.23	0.3	0.77	0.3
Y5-10	0.43	0.001	0.57	0.001
Y8–Y10	0.35	0.001	0.65	0.001
Fungi				
Y1–Y3	0.49	0.001	0.51	0.001
Y1–Y5	0.74	0.1	0.26	0.1
Y1–Y8	0.46	0.001	0.54	0.001
Y1–Y10	0.56	0.001	0.44	0.001
Y3–Y5	0.58	0.1	0.42	0.1
Y3–Y8	0.30	0.2	0.69	0.2
Y3–Y10	0.52	0.001	0.47	0.001
Y5–Y8	0.29	0.1	0.70	0.1
Y5-10	0.57	0.1	0.43	0.1
Y8–Y10	0.31	0.001	0.68	0.001

**Table A6:** The relative abundance (%) of soil bacterial and fungal community at phylum levels under different treatments.

**Dominant Bacteria Phylum**	**1Y**	**3Y**	**5Y**	**8Y**	**10Y**
Proteobacteria	21.9 ± 3.0 a	27.3 ± 1.0 ab	26.3 ± 2.0 ab	28.9 ± 1.0 b	31.0 ± 1.0 b
Actinobacteriota	9.7 ± 1.0 a	13.9 ± 1.0 a	12.9 ± 1.0 a	12.1 ± 1.0 a	10.6 ± 1.0 a
Acidobacteriota	15.5 ± 1.0 a	11.4 ± 1.0 b	13.0 ± 1.0 ab	11.8 ± 1.0 b	11.7 ± 1.0 b
Chloroflexi	3.4 ± 0.0 a	3.4 ± 0.0 b	3.3 ± 0.0 ab	3.4 ± 0.0 ab	3.4 ± 1.0 ab
Crenarchaeota	5.9 ± 3.0 a	0.7 ± 0.0 a	1.2 ± 0.0 a	1.5 ± 0.0 a	3.1 ± 1.0 a
Bacteroidota	2.6 ± 0.0 a	2.1 ± 0.0 a	2.2 ± 0.0 a	2.5 ± 0.0 a	2.6 ± 0.0 a
Myxococcota	1.9 ± 0.0 a	2.4 ± 0.0 a	2.3 ± 0.0 a	2.7 ± 0.0 a	1.9 ± 0.0 a
Gemmatimonadota	2.4 ± 0.0 a	2.9 ± 0.0 a	2.1 ± 0.0 a	2.2 ± 0.0 a	1.6 ± 0.0 a
Firmicutes	2.4 ± 1.0 a	2.1 ± 0.0 a	3.4 ± 1.0 a	1.7 ± 1.0 a	0.9 ± 0.0 a
Verrucomicrobiota	1.5 ± 0.0 a	1.9 ± 0.0 a	2.1 ± 0.0 a	2.4 ± 0.0 a	2.2 ± 0.0 a
Nitrospirota	1.9 ± 0.0 a	1.0 ± 0.0 a	1.2 ± 0.0 a	1.3 ± 0.0 a	1.1 ± 0.0 a
**Dominant Fungal Phylum**	**1Y**	**3**Y	**5Y**	**8Y**	**10Y**
Ascomycota	68.6 ± 5.0 a	76.3 ± 4.0 a	88.6 ± 2.0 a	59.4 ± 14 a	73.8 ± 6 a
Basidiomycota	8.0 ± 6.0 a	4.6 ± 0.9 a	2.9 ± 1.0 a	24.9± 17.0 a	6.0 ± 3.0 a
Mortierellomycota	3.5 ± 0.0 a	2.9 ± 1.0 a	2.9 ± 0.0 a	3.3 ± 1.0 a	7.1 ± 3.0 a
Mucoromycota	9.6 ± 1.0 a	2.6 ± 2.0 b	0.0 ± 0.0 b	0.0 ± 0.0 b	0.0 ± 0.0 b

The value is mean ± standard error (*n* = 3), and different lowercase letters in the same line indicate significant difference between treatments (*p* < 0.05).

**Table A7:** Full names of bacterial KEGG pathways and fungal functional guilds corresponding to the letter codes in Figure 8.

**Code**	**Full Name**
Bacterial KEGG pathways (level II)	
A1	Amino acid metabolism
A2	Carbohydrate metabolism
A3	Energy metabolism
A4	Lipid metabolism
A5	Nucleotide metabolism
A6	Metabolism of cofactors and vitamins
B1	Folding, sorting and degradation
B2	Replication and repair
B3	Translation
C1	Membrane transport
C2	Signal transduction
Fungal functional guilds (FUNGuild)	
A	Undefined saprotroph
B	Unassigned
C	Dung_Saprotroph-Undefined_Saprotroph-Wood_Saprotroph
D	Plant_Pathogen
E	Endophyte-Plant_Pathogen-Wood_Saprotroph
F	Plant pathogen–soil saprotroph–wood saprotroph
G	Plant pathogen–wood saprotroph
H	Animal pathogen–soil saprotroph
I	Endophyte–plant pathogen
J	Orchid mycorrhizal–plant pathogen–wood saprotroph
